# Synthetic patient and interview transcript creator: an essential tool for LLMs in mental health

**DOI:** 10.3389/fdgth.2025.1625444

**Published:** 2025-09-11

**Authors:** Aleyna Warner, Jeffrey LeDue, Yutong Cao, Joseph Tham, Timothy H. Murphy

**Affiliations:** ^1^ Department of Psychiatry, Faculty of Medicine, University of British Columbia, Vancouver, BC, Canada; ^2^ Djavad Mowafaghian Centre for Brain Health, University of British Columbia, Vancouver, BC, Canada; ^3^ BC Neuropsychiatry Program, Faculty of Medicine, University of British Columbia, Vancouver, BC, Canada

**Keywords:** LLM, python, psychiatry, large language models, synthetic data, automation

## Abstract

Developing high-quality training data is essential for tailoring large language models (LLMs) to specialized applications like mental health. To address privacy and legal constraints associated with real patient data, we designed a synthetic patient and interview generation framework that can be tailored to regional patient demographics. This system employs two locally run instances of Llama 3.3:70B: one as the interviewer and the other as the patient. These models produce contextually rich interview transcripts, structured by a customizable question bank, with lexical diversity similar to normal human conversation. We calculate median Distinct-1 scores of 0.44 and 0.33 for the patient and interview assistant model outputs respectively compared to 0.50 ± 0.11 as the average for 10,000 episodes of a radio program dialog. Central to this approach is the patient generation process, which begins with a locally run Llama 3.3:70B model. Given the full question bank, the model generates a detailed profile template, combining predefined variables (e.g., demographic data or specific conditions) with LLM-generated content to fill in contextual details. This hybrid method ensures that each patient profile is both diverse and realistic, providing a strong foundation for generating dynamic interactions. Demographic distributions of generated patient profiles were not significantly different from real-world population data and exhibited expected variability. Additionally, for the patient profiles we assessed LLM metrics and found an average Distinct-1 score of 0.8 (max = 1) indicating diverse word usage. By integrating detailed patient generation with dynamic interviewing, the framework produces synthetic datasets that may aid the adoption and deployment of LLMs in mental health settings.

## 1 Introduction

Recent research has explored LLM applications in healthcare, ranging from clinical decision support and medical education to administrative automation (1, 2). In psychiatric settings, LLMs have been investigated for risk assessment and predictive analytics, helping identify high-risk patients and potential complications before they escalate. They have also been used to tag and summarize patient behaviors during clinical interviews, offering structured insights that assist clinicians in diagnoses or provide secondary perspectives (3, 4). Beyond decision support, patient-facing applications such as symptom assessment chatbots have been introduced to offer preliminary guidance; however, these tools require careful human oversight to ensure accuracy and prevent misinterpretations, particularly in high-risk mental health scenarios (2). Despite these advancements, there are challenges that limit the effectiveness of LLMs in mental health applications. A major barrier is the lack of diverse, high-quality training data, as psychiatric assessments involve complex, context-dependent interactions that are difficult to standardize. Additionally, privacy regulations—such as HIPAA, GDPR, and PIPEDA—impose strict guidelines on the collection, storage, and use of personal health data, making it challenging for researchers to access diverse and representative datasets for model training. To address this, synthetic data generation has been proposed as a privacy-compliant solution, as they allow researchers to develop and test models without accessing sensitive information (5, 6). This approach has been particularly valuable in specialty fields such as oncology, neurology, and cardiology, where patient datasets are often limited due to privacy concerns and disease rarity (5). However, while synthetic data has addressed some challenges in these fields, its application in mental health research remains an open area for exploration, particularly for generating adaptive, context-aware interactions.

To address this, we present a synthetic patient and interview generation framework that leverages two instances of Llama 3.3 70B models. Our objective is to comprehensively address key challenges in deploying AI within mental health settings by:

• **Facilitating Dynamic Interactions:** The framework simulates [psychiatric] intake assessments by assigning distinct roles: one model acts as the interviewer and conducts the structured, adaptive interviews, while the other model acts as the patient and generates responses based off of a generated profile. This design mirrors the interactive dynamics of real-world clinical settings, allowing for natural and responsive exchanges.
• **Generating Realistic Profiles:** A separate component within the framework is responsible for generating synthetic patient profiles by combining predefined demographic and clinical variables with dynamically produced content, ensuring diversity and realism.
• **Providing Customizability:** The framework allows users to tailor question banks and patient parameters to align with specific research, training, or educational objectives. This flexibility enhances its applicability across various mental health contexts.
• **Ensuring Versatility:** By combining detailed profile generation with interactive interviews, the tool addresses a range of potential applications, including testing LLM capabilities, creating training materials, exploring hypothetical scenarios, or facilitating fine-tuning efforts for domain-specific applications.

This framework represents a novel, resource-efficient, and privacy-respecting approach to generating synthetic data and interactions, addressing critical challenges in mental health research and tool-development.

## 2 Methods

### 2.1 System architecture

**Figure 1:**
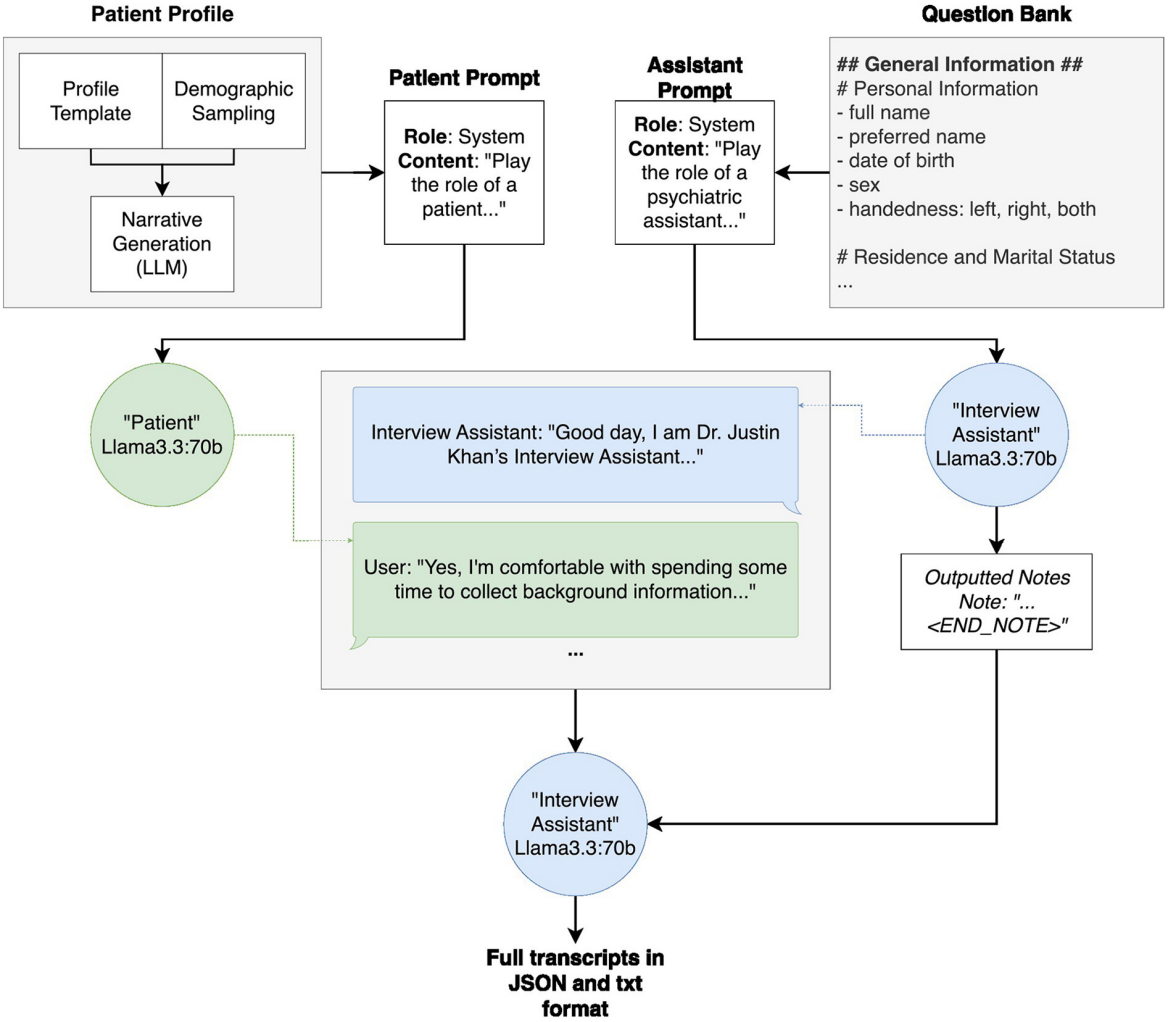
Overall system architecture of patient profile generation, interview generation and output. The patient profile generation, which is further broken down in Figure 2, takes in a profile template, fills in demographic attributes using random sampling based on the population statistics, and subsequently fills in narrative details (for example, the names and ages of any children) using an instance of Llama3.3:70b. The patient profile is then inserted into the “patient prompt” prior to the transcript generation. Similarly, the question bank is inserted into the interview assistant model's “assistant prompt” prior to the transcript generation. During transcript generation, these prompts are passed to their respective models, and the models are called back and forth, the “interview assistant” model asking questions from the question bank and the “patient” model answering with information from the patient profile. Throughout the interview, the “interview assistant” model summarizes the “patient” responses. Once all questions have been asked and answered, the “interview assistant” model outputs both the interview transcript and summary notes into one document, in both JSON and.txt format.

The methodology employed in developing and implementing the LLM-driven psychiatric interview system ensures structured yet adaptive interviews using two instances of Llama 3.3-70B models (Figure 1). We have included software code for generating synthetic patient and interview transcripts on a public repository https://github.com/ubcbraincircuits/SPIT_Generation.git. One instance functions as the Interviewer Model, responsible for dynamically selecting and delivering structured interview questions, while the other instance serves as the Patient Model, generating responses based on predefined profile data and free-text generation. Unlike large-scale AI deployments that require high-performance computing clusters, this system operates efficiently on a locally run setup with a modest GPU configuration, demonstrating that sophisticated LLM-driven psychiatric assessments can be conducted without the need for extensive computational infrastructure. This approach ensures accessibility, lower operational costs, and full control over data security while maintaining a balance between structured clinical assessment and adaptive conversational flow, enabling more naturalistic and contextually relevant responses.

#### 2.1.1 Patient profile generation

**Figure 2:**
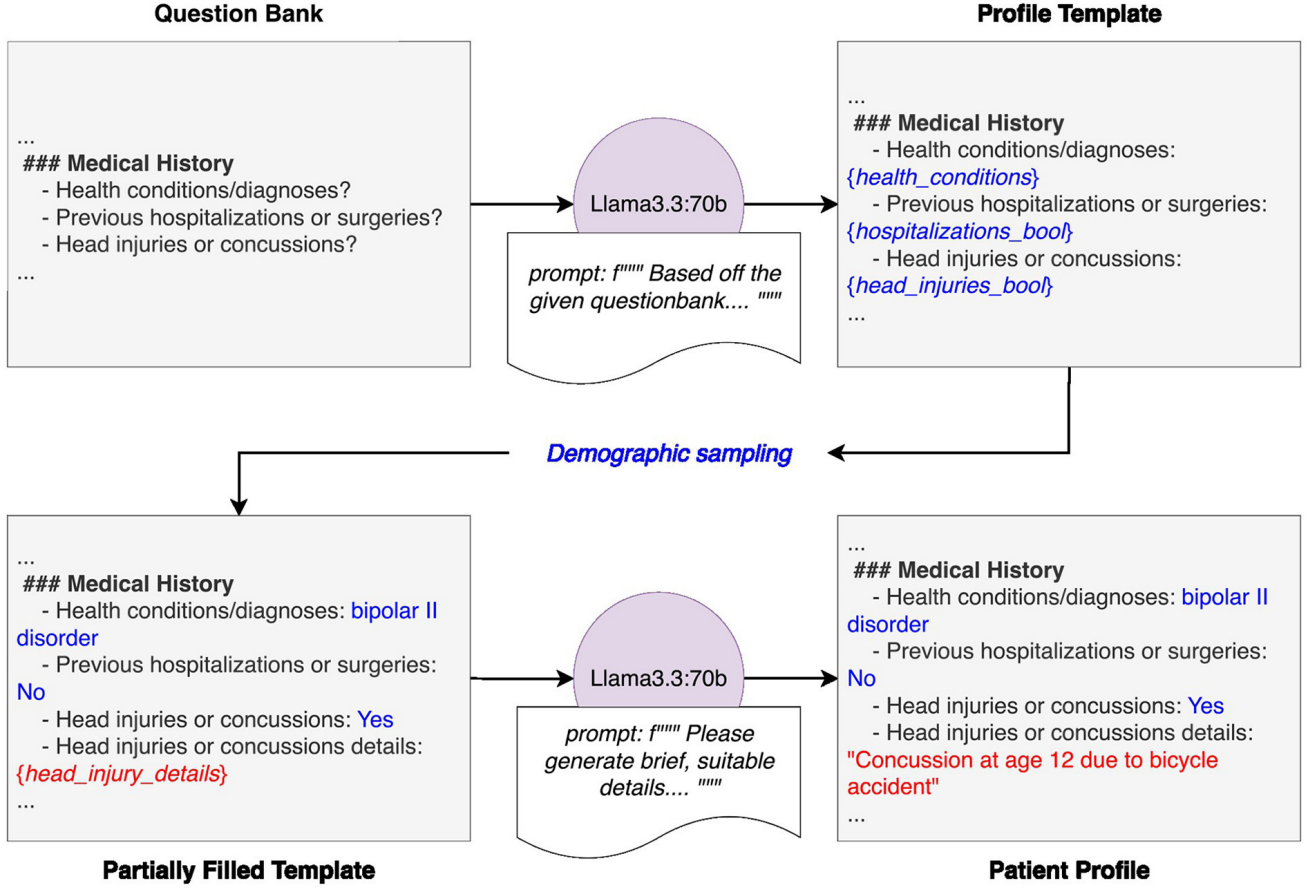
Detailed diagram of patient generation system. The system begins with a predefined question bank, which is provided to an instance of Llama3.3-70B along with a prompt. This prompt instructs the model to generate a patient profile template that strictly adheres to the question bank's structure and format, reinforced with an illustrative example. Demographic attributes are then assigned to the template through random sampling based on population statistics. The partially completed profile is then passed to another instance of Llama3.3-70B with a prompt directing it to fill in the remaining sections with realistic but entirely fictional details. The final output is a structured, fully populated patient profile ready for use in downstream applications.

A locally run Llama 3.3-70B model generates synthetic patient profiles (Figure 2) following predefined structures, ensuring consistency while allowing for dynamic variability. To develop the original patient profile template, a predefined question bank was provided to an instance of Llama3.3:70b, along with a prompt. This prompt instructed the model to generate a structured patient profile that strictly adhered to the question bank's format, reinforced with an illustrative example. This approach ensured that the template aligned with the intended structure before being manually reviewed and refined for integration into the system. This structured template is then populated with demographic attributes based on real-world statistics before a final LLM instance further enriches the profile with narrative details (Figure 2). These profiles incorporate fixed demographic variables—such as age, gender, and medical history—derived from real-world population distributions relevant to the authors' geographic region, enhancing applicability to local healthcare contexts. The demographic distributions are informed by publicly available datasets, including Statistics Canada, the British Columbia (BC) Ministry of Education and Families, the BC Ministry of Health, and the Vanier Institute of the Family (7–29).

To enhance realism, we employ the Faker Python package to generate names, dates of birth, addresses, and occupations (30). However, unlike conventional usage where names are sampled at random, our approach weighs Faker's ethnic name distributions according to regional census data. This ensures that name assignments align with realistic demographic proportions.

Additional characteristics are assigned based on curated lists and dictionaries stored in a variables.py file. These include attributes that are not constrained by demographics, either by choice or lack of data, such as allergies, health supplements, recreational drug use, reasons for doctor appointments, and psychiatric conditions with corresponding treatments. For instance, psychiatric conditions are drawn from a dictionary where conditions serve as keys and their commonly prescribed medications (with dosages) are listed as values. This dictionary was initially generated using ChatGPT to compile common psychiatric conditions and associated medications, then manually reviewed and refined. The appearance rate of these conditions are also randomized, as it is not uncommon for a patient to have comorbid diagnoses, so we include a chance that one to three psychiatric conditions are given to a patient. Certain characteristics, such as this one, remain fully randomized due to a lack of reliable demographic data. While they currently follow a predefined probability distribution, they can be adjusted in the future as relevant population statistics become available.

Once the structured patient data is established, another instance of Llama3.3:70b generates expanded descriptions for key aspects such as family and social history, health and injury history, personal interests, and daily lifestyle patterns. This step ensures that synthetic patients exhibit diverse and more natural storytelling, increasing their utility for training and research purposes. The LLM is guided by a structured prompt that instructs it to incorporate details while maintaining logical consistency with the assigned attributes.

All patient profiles are stored in a structured format, ensuring easy retrieval and modification. The patient generation is implemented in “llm_patient_creator.ipynb” Jupyter Notebook within the project repository (https://github.com/ubcbraincircuits/SPIT_Generation.git). Users can modify variables.py to update predefined lists or dictionaries and adjust probability distributions. Additionally, patient records can be manually edited post-generation to refine specific cases before use in downstream applications.

#### 2.1.2 Prompt design

The Interviewer and Patient Model prompts are built around a structured Chain-of-Thought prompting strategy inspired by the INSTRUCT framework (8), emphasizing stepwise task decomposition, logical consistency, and grounded output formatting. This methodology guides each model through the structured yet dynamic process of conducting and responding to a psychiatric interview, ensuring coherence, traceability, and grounded dialogue generation.

Both prompts begin with context paragraphs establishing clear roles: the Interviewer Model collects detailed psychiatric information in a safe, ethical, and non-judgmental manner, setting a clinical yet supportive tone; the Patient Model simulates a synthetic patient participating realistically within the defined scenario, maintaining character consistency and avoiding breaking the fourth wall. These context paragraphs orient each model clearly, embedding professional and ethical interaction norms.

Following this introduction, structured sections enable targeted interactions:

• The Interviewer Model uses a tagged section presenting the current set of interview questions. This modular approach ensures comprehensive coverage of structured interview domains.
• The Patient Model uses a <PATIENT_INFO> tagged section detailing a synthetic patient profile, encompassing structured attributes (e.g., demographics, diagnoses) and freeform traits (e.g., emotional tone, life events), ensuring responses remain character-consistent and contextually grounded.

Each model's Guidelines section supports natural-sounding, empathetic, and variable interactions. For the Interviewer Model, varied phrasings for acknowledgments, clarification prompts, and transitions are provided to avoid repetitive language, particularly regarding sensitive topics like trauma or substance use. Explicit examples model respectful handling and graceful topic shifts. The Patient Model's Guidelines encourage behavioral variability, instructing the model to express realistic emotional tones, occasional vagueness, mild contradictions, and natural handling of uncertainties or refusals.

At the core of both prompts is a structured INSTRUCT-based Chain-of-Thought reasoning loop:

Interviewer Model (Six-step loop):

1. **Identify the Previous Question** asked by the model itself, ensuring continuity.
2. **Summarize the Patient's Latest Answer** using a structured note format (Note: … <END_NOTE>) for downstream parsing and traceability.
3. **Assess Completeness** based on predefined criteria: clarity, detail, and relevance.
4. **If Clarification is Needed**, prompt for it using varied polite phrasings, skipping all other steps.
5. **If All Questions Have Been Asked**, return RESPONSE: <STOP> to indicate interview completion.
6. Otherwise, compose the next turn by integrating an appropriate acknowledgment and posing the next question from the queue—ensuring variation in phrasing and emotional sensitivity.

Patient Model (Five-step loop):

1. **Identify the Most Recent Question** asked by the interview assistant, ensuring that the response is directly relevant to what was asked.
2. **Retrieve Relevant Information** from the patient profile, focusing on key dimensions such as timelines, frequency, or severity.
3. **Formulate a Response** that draws on these details, staying brief, character-consistent, and focused on the core of the interview assistant's question.
4. **Check for Clarity**, adjusting vague answers with an additional emotional or temporal cue if necessary.
5. **Deliver the Final Answer**, always beginning with RESPONSE: to maintain output consistency and compatibility with the surrounding system.

Both loops clearly separate internal reasoning from conversational outputs, with only the final RESPONSE: communicated between models. Embedded example workflows illustrate behavior in common edge cases (e.g., vagueness, refusal, emotional distress), reinforcing consistency and sensitivity.

This combined prompt design blends structured INSTRUCT-style reasoning with realistic dialogue generation, enabling comprehensive data collection and rapport-oriented interaction while supporting controlled simulation realism. These prompts are stored in the files “assistant_prompt_v6.txt” and “patient_prompt_v2.txt”, and implemented in the “double_model_chunking_ollama.py” file available in the project GitHub repository (https://github.com/ubcbraincircuits/SPIT_Generation.git).

#### 2.1.3 Question bank

Transcript generation was created in collaboration with a psychiatrist, who provided a mock interview transcript and an outpatient intake questionnaire. We used these documents as the basis for questions that should be covered in the synthetic interview by extracting topics into a question bank. The final question bank consisted of 47 points with 5 sections titled General Information, Medical History, Family History, Personal History, and Additional Comments. The question bank is used by the interview assistant to provide structure to its question-asking. We found that it was necessary to add specific follow-up points in the question bank itself, and provide a final point for ending the interview. The complete question bank is provided in the “questionbank_chunked.txt” file in the project Github (https://github.com/ubcbraincircuits/SPIT_Generation.git).

#### 2.1.4 Transcript generation

##### 2.1.4.1 Interview structure and flow

**Figure 3:**
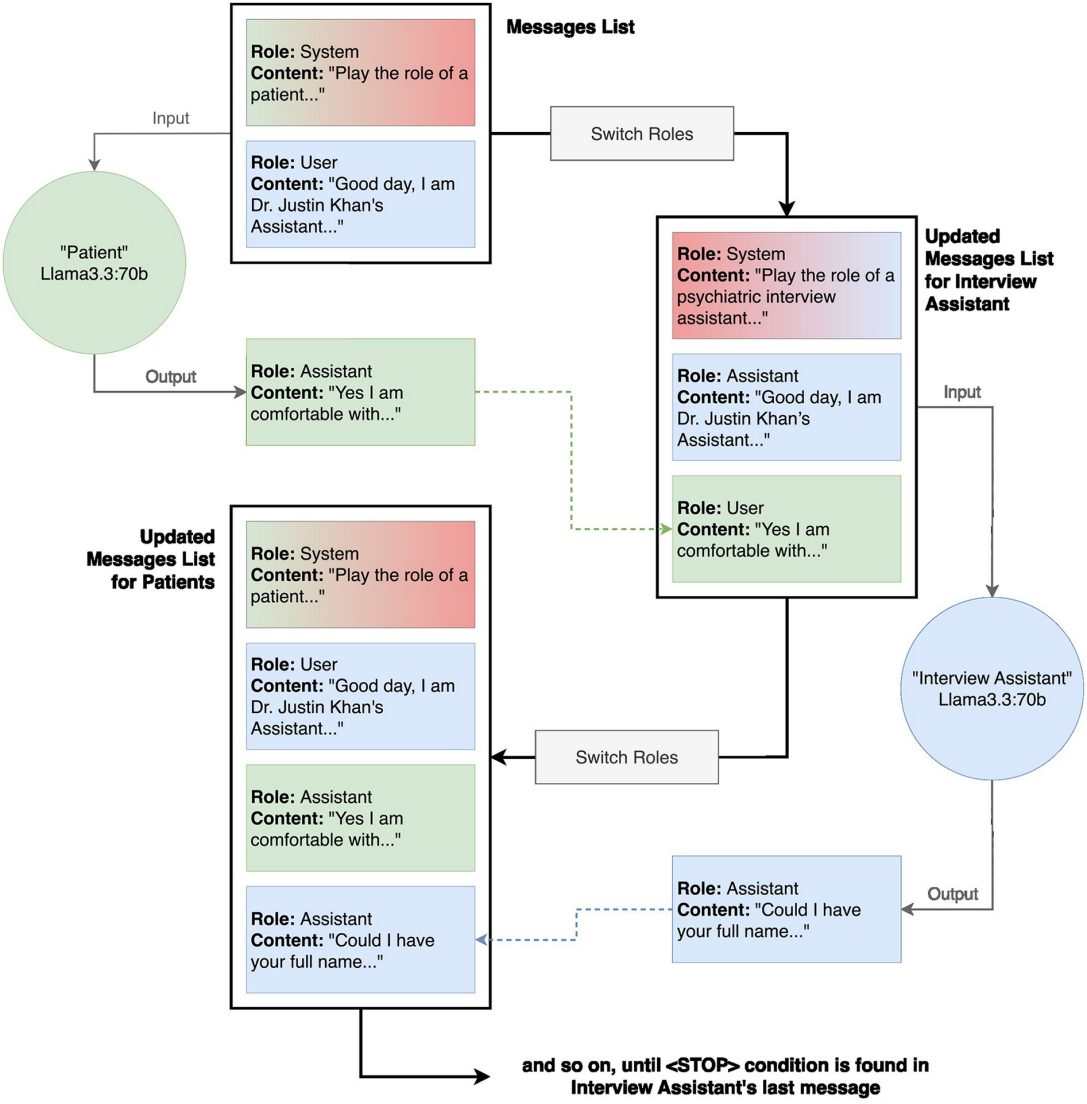
Detailed code architecture of transcript generation. The transcription generation method uses two instances of Llama3.3:70b, one acting as the “Patient” and the other as the “Interview Assistant”. To maintain clear conversational roles, the patient model receives its own responses tagged as “interview assistant” and the interview assistant's questions tagged as “user” in the updated messages lists, while the interview assistant model follows the reverse role assignment. The process begins with a system message providing the patient prompt, followed by an initial user message to start the interaction. The patient model generates a response, which is appended to the conversation history. The roles are then inverted for the interview assistant model, ensuring that its responses align with the expected dialogue structure. This iterative loop continues until a predefined stop condition is met, signaling the conclusion of the interview.

The interview process follows a structured, looped flow in which two instances of Llama 3.3-70B are running in tandem—one acting as the Interview Model and the other as the Patient Model—exchange messages until a built-in stop condition signals the end of the session (Figure 3). It begins with the Patient Model receiving the hard-coded starting question and generating its initial response. The Interview Model then ingests that reply alongside the full, pre-defined question bank and produces a single, combined acknowledgment-and-question output, ensuring that each turn feels conversational yet remains faithful to the scripted prompts. The models simply alternate patient responses and interview questions—while the Interview Model records key clinical details after each exchange—until the stop condition is triggered.

##### 2.1.4.2 Turn management

To keep turns coherent and prevent role confusion, every message is explicitly tagged and roles are swapped on each pass. When querying the Patient Model, its own answers are labeled “assistant” and the Interview Assistant Model's questions “user”. Before the next turn, we reset the system prompt to the Interview Model's template, relabel the patient's last reply as “assistant”, and queue up the next question as “user”. This append-and-swap cycle begins with the Patient Model prompt as the first system message and the starter question as the first user message—and repeats until the Interview Model detects its stop-condition token.

##### 2.1.4.3 Chunking and dynamic question loading

To optimize efficiency, the system employs a chunking and dumping mechanism, structuring the question bank into predefined subcategories based on headers in the input text file. Our question bank, derived from an actual intake question bank used in a clinic in Vancouver and reflective of other common clinical interviews (i.e., SCID-5), is delivered in structured chunks as the interview progresses. The system dynamically loads a new chunk just as the Interviewer Assistant Model is about to ask the final question of the current chunk, preventing interruptions or premature termination due to a lack of available questions. This preemptive loading mechanism ensures a fluid and complete interview experience while maintaining logical continuity. Once a chunk is completed, previous interactions—both questions and responses—are removed from active context and archived in the transcript. This approach optimizes GPU usage, prevents early termination, and ensures that only relevant information remains in active processing, allowing the interview to proceed smoothly without exceeding system constraints.

##### 2.1.4.4 Limits and trade-offs

To enforce correct turn-taking, every message is explicitly tagged and roles are swapped on each pass; the Interviewer Model emits a <STOP> token when it has asked all questions. Sessions that fail to produce <STOP> or otherwise violate these role tags are flagged as prompt-adherence failures and reviewed manually (see Results 3.2). We do not currently include automated checks for hallucinations, incoherence, or role deviation, but such quality-control mechanisms (e.g., classifier-based consistency validators) could be integrated as a future enhancement (see Discussion 4.4). Because at most one 6,144-token chunk (plus a brief seeded summary of previous dialogue) resides in the model's active window, neither model has direct access to the full conversation history. This design keeps computational requirements modest and avoids token-overflow, but can introduce subtle gaps in long-range coherence at chunk boundaries. In a higher-capacity deployment—e.g., on GPUs with larger memory or models architected for extended context windows—one could retain the entire dialogue in memory to further enhance narrative continuity. We also deliberately calibrate variability vs. consistency by tuning our sampling parameters (temperature, top-k/top-p) and embedding strict profile tags in every prompt (see Results 3.2). This ensures patient replies remain true to their assigned character while still exhibiting natural diversity.

##### 2.1.4.5 Summary generation and implementation details

At interview completion, the Interviewer Assistant Model determines when all relevant questions have been asked and generates a structured summary based on the recorded notes. This summary synthesizes key details from the interview, providing a concise yet comprehensive overview of the patient's responses. The transcript generation process is called in the “create_transcripts.ipynb” file and implemented in the “double_model_chunking_ollama.py” file in the project Github (https://github.com/ubcbraincircuits/SPIT_Generation.git).

#### 2.1.5 Inference configuration

All calls to our two Llama 3.3:70B instances (Interviewer and Patient models) use the following Ollama client settings:

• **temperature** **=** **0.9:** Balances creativity and coherence: a value near 1.0 allows for varied phrasings and richer narrative detail (e.g., different acknowledgment styles), while avoiding the randomness that would emerge at values closer to 1.0+ (31–34).
• **top_k** **=** **40:** Truncates the sampling pool to the 40 most likely tokens at each generation step, reducing the risk of extremely low-probability (“off-topic”) words while still preserving enough options for lexical diversity (31–34).
• **top_*p*** **=** **0.9:** Implements nucleus sampling by including only the smallest set of tokens whose cumulative probability reaches 90%. This dynamically adjusts the sampling set to the model's confidence distribution, combining coherency with variability (31–34).
• **num_ctx** **=** **6,144:** Allocates a 6,144-token context window to support long, multi-turn interviews. To prevent context overflow, we employ the chunking-and-dumping mechanism described in Section 2.1.4, which offloads completed question–answer chunks from active memory before appending new prompts.

These parameters were chosen based on best practices from the literature to balance adherence to our structured prompts—so that question order and profile grounding remain accurate—while still providing the variability needed for a natural, empathetic conversational style.

### 2.2 System implementation and performance

The system is deployed locally on a dedicated desktop workstation running Ubuntu 22.04.5 LTS (kernel version 6.8.0–51-generic), featuring an AMD EPYC 7402P 24-Core Processor paired with 256 GB of DDR4 ECC RAM. The computational demands of the Llama 3.3-70B models are met by two NVIDIA RTX 4090 GPUs, configured with NVIDIA driver version 550.144.03 and CUDA 12.4. Model inference is managed through Ollama (version 0.5.7), an open-source platform specifically designed to streamline the deployment and operation of large language models.

This locally-hosted environment ensures data security—crucial for potential applications involving sensitive patient information—as well as reduced latency and complete control over computational resources. By eliminating reliance on external cloud services, the system guarantees consistent performance, privacy, and precise execution tailored specifically to facilitate future secure clinical applications. However, if privacy is not a primary concern, the model can alternatively be deployed using cloud-based services on systems without GPU hardware.

## 3 Results

### 3.1 System architecture and design

Our synthetic patient generation framework consists of three key stages—patient profile generation, interview simulation, and output formatting (see Figure 1). In this framework, patient profiles are generated via a templating system, populated with demographic attributes through probabilistic sampling, and further enriched with narrative content using an instance of Llama3.3:70B. As described in the methods, during transcript generation, the interview assistant model queries the patient model using a curated question bank, simulating a naturalistic interview. The resulting interaction is compiled into a transcript and accompanying summary, exported in both.txt and JSON formats.

#### 3.1.1 Patient profile generation pipeline

The patient profile generation pipeline begins with a question bank and a guiding prompt passed to Llama3.3:70B, instructing the model to generate a structured template that adheres to the question framework (see Figure 2). Demographic variables were then sampled from real-world population statistics to partially complete the profile (7–29). A second instance of Llama3.3:70B fills in narrative fields with plausible yet entirely fictional details. This process resulted in a structured, demographically grounded, and narratively rich profile ready for downstream use. Using the indicated hardware it took about 3 h to generate 1,000 patients and ∼50 h to generate 100 transcripts.

#### 3.1.2 Transcript generation workflow

The conversation between patient and interview assistant models was orchestrated through a dynamic role-based messaging system (see Figure 3). Two instances of Llama3.3:70B were used: one for the “Patient”, the other for the “Interview Assistant”. To preserve turn-taking realism, each model receives reversed role labels in their message histories. The interview assistant model asks questions from the question bank, while the patient model generates responses based on its profile. The loop continued until a predefined stop condition was met, producing a transcript and a structured set of summary notes and attributes of the patient. Example transcripts are available on the Github Repository in both.JSON and.TXT format. (https://github.com/ubcbraincircuits/SPIT_Generation/tree/main/transcript_generation/transcripts/llama3.3/DM).

### 3.2 Metrics and statistical analysis

To systematically quantify our synthetic data's linguistic richness, redundancy, and demographic fidelity, we employ three well-established metrics. Below, each metric is defined in detail, including its mathematical formulation, interpretive range, and relevance to our analyses.

• **Distinct-1** (35) is a lexical diversity metric that measures the proportion of unique unigrams (single-word tokens) in generated responses. Distinct-1 ensures that the model produces varied and naturalistic language rather than reusing the same wording or phrases across different patient profiles. Formally:
  $$Distinct\;1\;=\;\frac{\#\;of\;unique\;unigrams}{Total\;\#\;of\;unigrams}$$

Values range from 0 to 1: a score of 0 indicates complete repetition (no unique tokens), while a score of 1 denotes perfect diversity (every token is unique). In our context, higher Distinct-1 scores reflect more varied, human-like language use. We apply this metric to patient narratives (3.2.2) and interview transcripts (3.2.3).

• **The Duplicate Ratio** quantifies redundancy by calculating the fraction of output segments that appear identically more than once. If $N_{dup}$ is the total count of duplicate segments and $N_{tot}$ the total number of segments, then:
  $$Duplicate\;Ratio\;=\frac{N_{dup}}{N_{tot}}\;$$

A low Duplicate Ratio (near 0) indicates minimal repetition, whereas higher values point to frequent rote responses. We use this metric to assess narrative fields (e.g., “work history”, “relaxation methods”) for unexpected uniformity (3.2.2).

• **χ^2^ Goodness-of-Fit Test** is used to evaluate whether our synthetic demographic distributions match real-world data. A *p*-value above 0.05 indicates no significant deviation. We apply this test to ethnicity, age, and disability categories (3.2.1) and relationship status and parental status by age/sex (3.2.1)

#### 3.2.1 Demographic validity of synthetic patients

**Figure 4:**
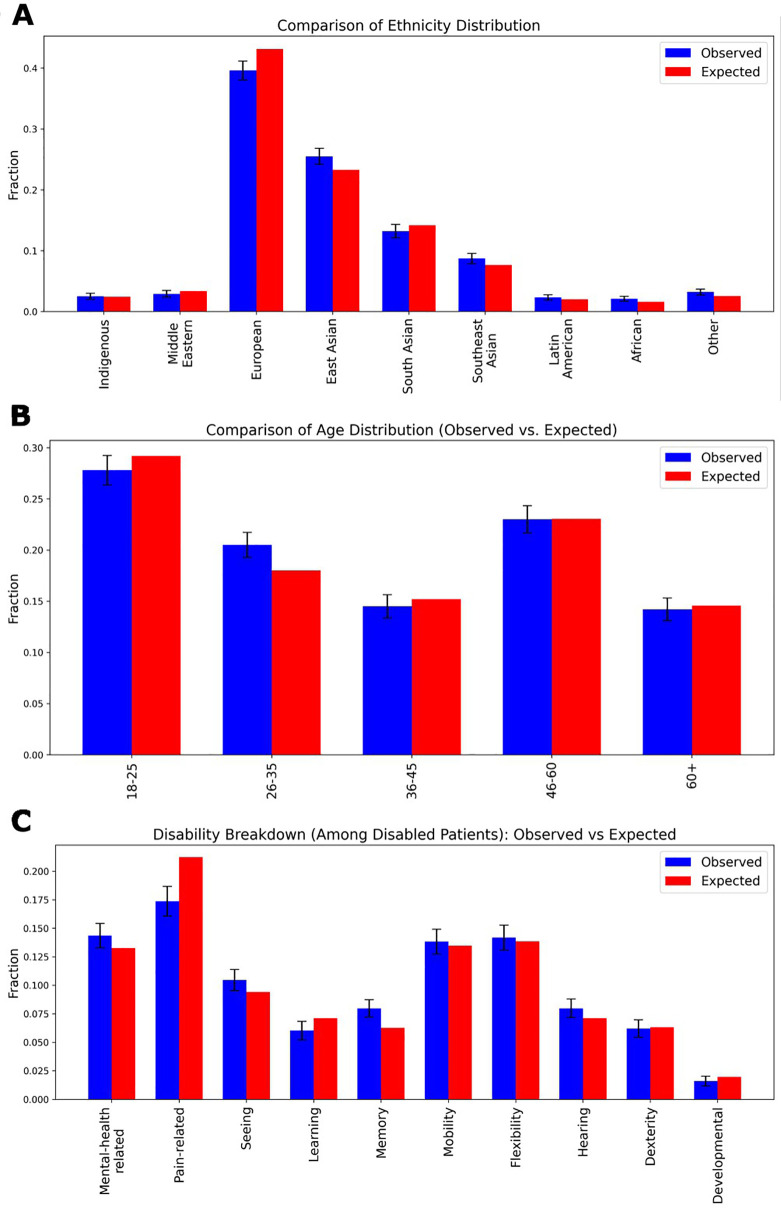
Demographic distributions of a One thousand patient sample. **(A)** The ethnicity distribution in the generated dataset compared to real-world demographic statistics from the authors’ local region. The observed distribution of ethnic groups in the synthetic patient dataset is plotted alongside expected proportions. A chi-square test yielded a value of χ^2^ = 11.90 with a *p*-value of 0.1556, indicating no significant difference between the generated and actual ethnic distributions. **(B)** The age distribution comparison, showing the proportion of different age groups in the generated dataset vs. the actual demographic distribution. A chi-square test yielded a value of χ^2^ = 2.47 with a *p*-value of 0.6495, again indicating no significant difference. **(C)** Expected values were computed using sex- and age-specific disability prevalence rates, with the total probability of being disabled distributed proportionally across ten categories: Mental-health related, Pain-related, Seeing, Learning, Memory, Mobility, Flexibility, Hearing, Dexterity, and Developmental disabilities. The observed data were derived from the frequency of assigned disability labels within the dataset. A chi-square test yielded a value of χ^2^ = 9.83 with a *p*-value of 0.3648, again indicating no significant difference. In all panels, standard deviation error bars represent the variance within the generated dataset.

To assess the validity of synthetic demographic information, we compared key demographic and health-related characteristics against regional population prevalence statistics (see Figure 4) (7–9). We evaluated ethnicity distributions in a one-thousand-patient sample against census data using a chi-square test (χ^2^ = 11.90, *p* = 0.1556), finding no significant difference (see Figure 4A). Figure 4B similarly examines age-group frequencies, again demonstrating close alignment with real-world age demographics (χ^2^ = 2.47, *p* = 0.6495). Expected distributions for ten disability categories were derived from published, age- and sex-specific prevalence rates: for each synthetic patient, we first determined whether they had any disability based on their gender and age bracket, then—using overall type-specific prevalence percentages—assigned them to a particular disability category (see Figure 4C). The observed frequencies in our generated cohort closely matched these expected proportions across all categories (χ^2^ = 9.83, *p* = 0.3648).

**Figure 5:**
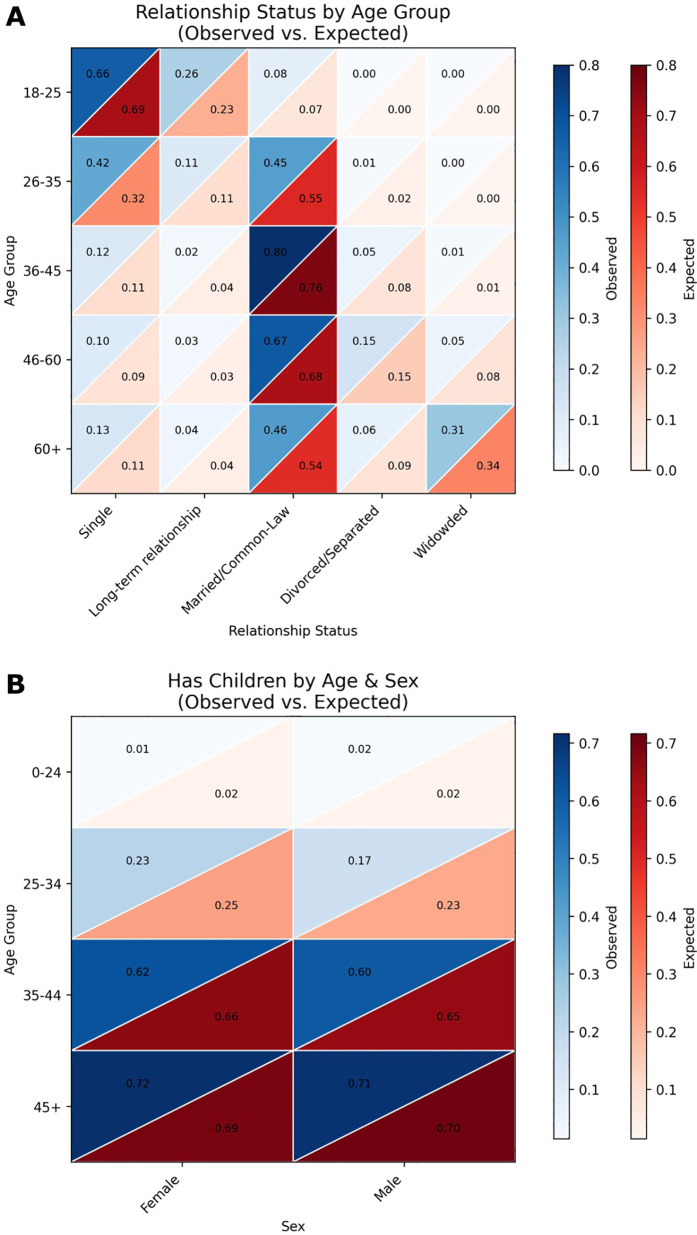
Heatmap comparisons of generated patient demographics and real-world population data. **(A)** Heatmaps depicting the distribution of relationship status across age groups in the generated patient dataset compared to real-world demographic data. Relationship categories include Single, Living-apart-together, Married, Common-law, Divorced/Separated, and Widowed. Each cell represents the proportion of individuals within a specific age and relationship status category. The alignment between the generated and real-world distributions was assessed using chi-squared tests, with a resulting χ^2^ = 12.24, *p* = 0.967. **(B)** Heatmaps showing the probability of having at least one child across age groups in the generated patient dataset vs. real-world data. Color intensity corresponds to the proportion of individuals with children within each age group. Statistical comparison of the distributions yielded a z-score of −0.6517 and a *p*-value of 0.2573.

Similarly, we compared the expected (based on the regional population prevalence statistics) relationship and parental status to that of the generated set of 1,000 patients. Results for relationship status by age and sex are shown in the heatmap in Figure 5A and for parental status in Figure 5B with blue denoting the synthetic patients and red the regional population prevalence statistics. Again, as with ethnicity, age, and disabilities, we used a chi-square test to determine whether the group of generated synthetic patients showed significant deviation from the regional population prevalence statistics (χ^2^ = 12.24, *p* = 0.967; indicating adherence to regional statistics).

#### 3.2.2 Narrative diversity in model outputs

**Figure 6:**
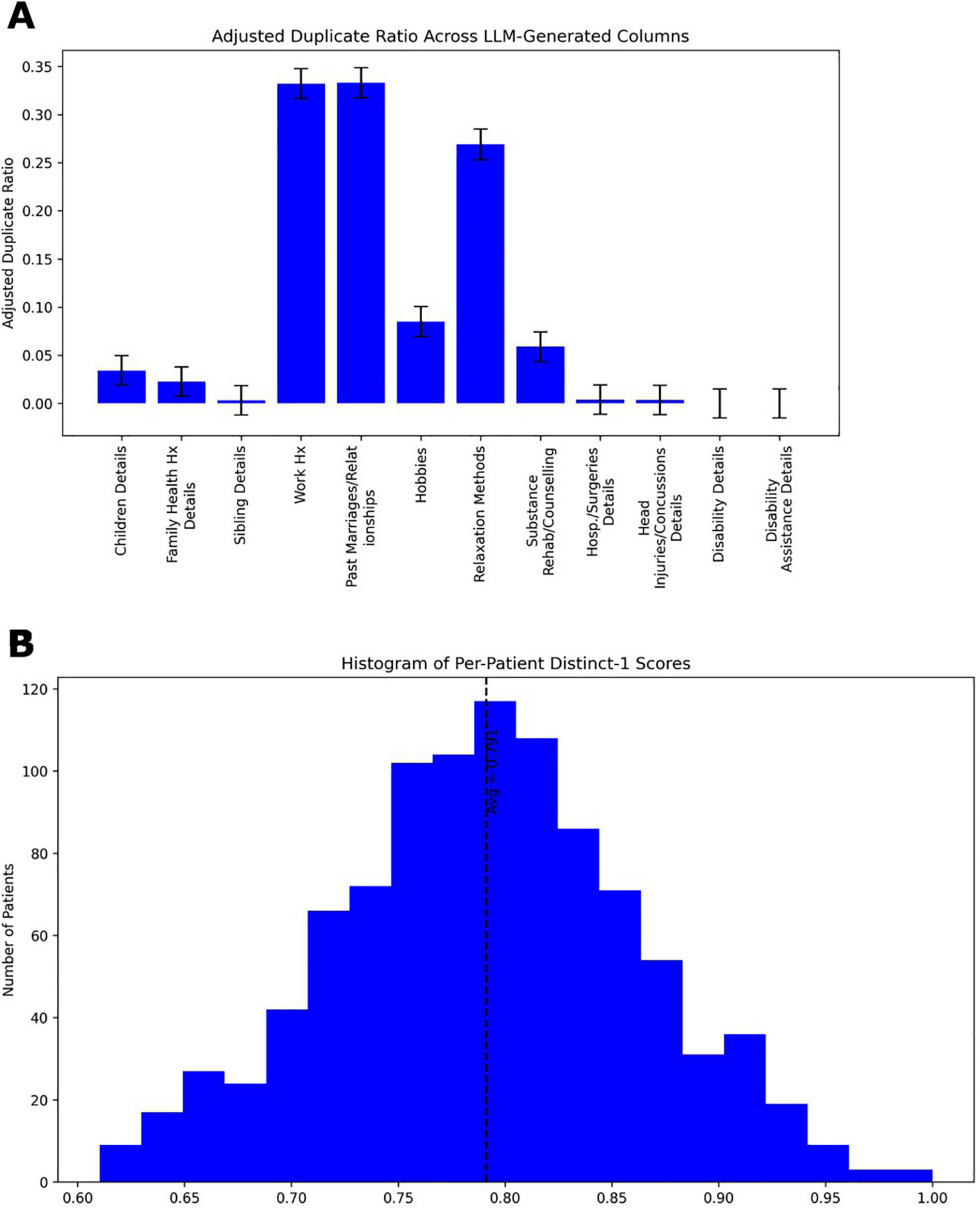
Patient generator LLM narrative data metrics. **(A)** Duplicate Ratio measures the proportion of completely identical responses generated by the LLM. A higher duplicate ratio indicates that the model frequently produces identical outputs across different runs (inferences), suggesting lower diversity. A lower duplicate ratio reflects greater variability in generated responses. **(B)** Histogram of Distinct-1 scores calculated on all LLM generated content for each patient. As noted elsewhere, Distinct-1 quantifies the diversity of unigrams on a scale of zero to one by calculating the fraction of unique unigrams relative to the total unigrams count. Higher Distinct-1 scores signify a broader vocabulary and greater lexical diversity, while lower scores indicate more repetitive word usage. The average Distinct-1 score across each transcript is 0.791 with a standard deviation of 0.070.

We analyzed the linguistic variability of generated patient narratives using two diversity metrics (Figure 6). We calculated the Duplicate Ratio metric (Figure 6A), which was low across most of the patients LLM-generated attributes, but notably higher in a few specific categories, namely “work history”, “past marriages/relationships” and “relaxation methods”, indicating some expected repetition in generated outputs. We also calculate a Distinct-1 score for all LLM generated content for each patient and present a histogram in Figure 6B. The histogram reflects a high ratio of unique unigrams, consistent with rich lexical diversity in the patient narrative details. Together, these results indicate that the model produces varied and, in most cases, non-redundant narrative details, enhancing the realism of the synthetic data.

#### 3.2.3 Transcript metrics

In the 79 transcripts that complied with the stop-condition protocol (see below), lexical diversity was notably higher in the patient's responses than in the interview assistant's responses. The median Distinct-1 score was 0.45 for user turns as compared to 0.33 for interview assistant turns. These results show the generally high adherence rate and some clear asymmetry in language variety between the patients and the interview assistant. For comparison, we also calculated the Distinct-1 score on a set of transcripts from a radio show comprising 10,000+ episodes and 3,000,000+ utterances (36). We calculated the Distinct-1 score per episode and averaging across episodes resulted in a score of 0.50 with a standard deviation of 0.11. Given the values reported above for Distinct-1 for our patient and interview assistant models, this indicates that lexical diversity of the patient model is within the range of normal human conversation. The interview assistant is lower due to the repetitive nature of answer acknowledgements (e.g., “Thank you for sharing that…”).

**Figure 7:**
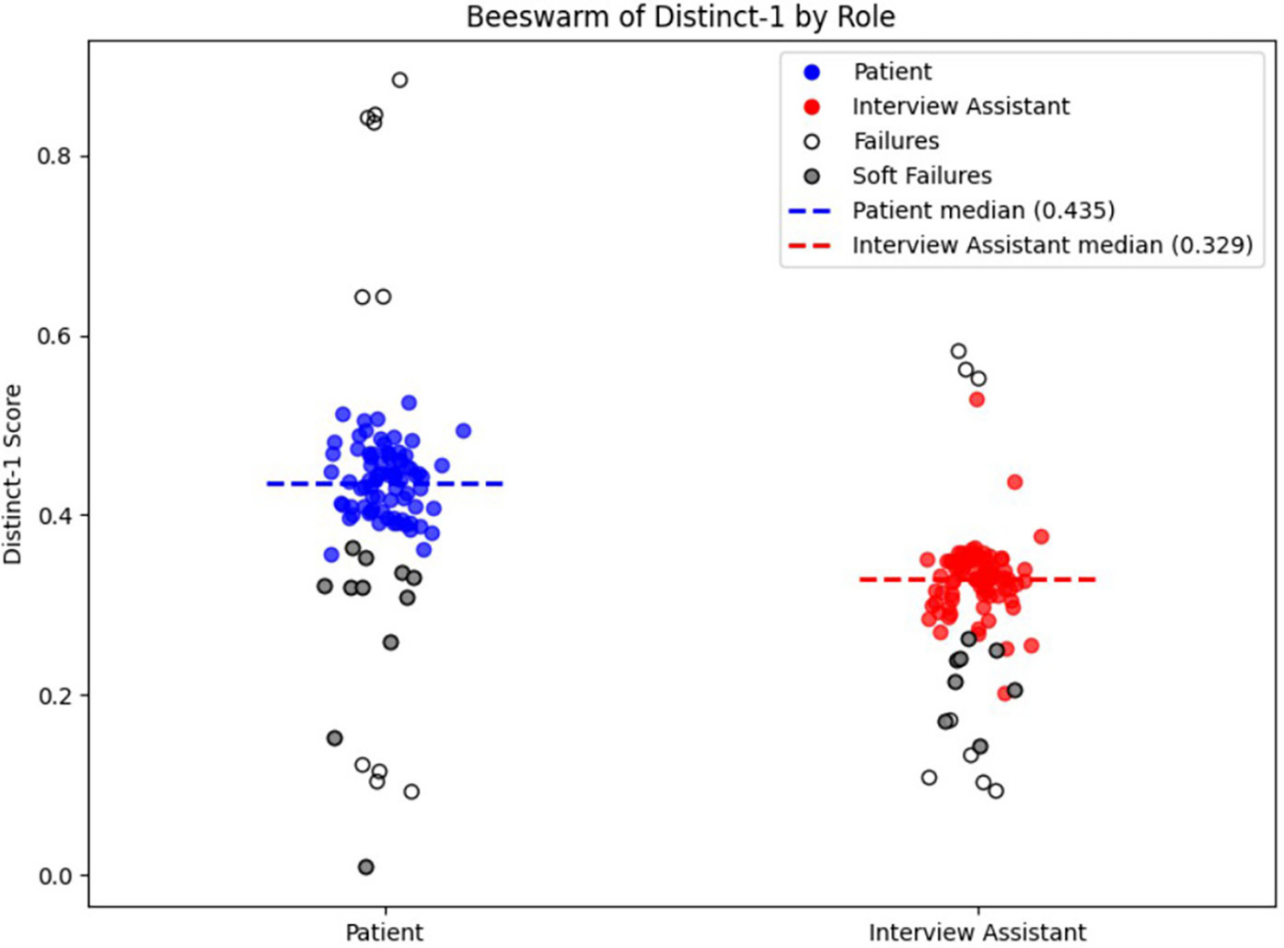
Distinct-1 score per transcript by role. The plot shows the Distinct-1 ratio—the proportion of unique unigrams to total tokens—computed for each transcript separately for patient (blue) and interview assistant (red) turns. Dashed horizontal lines mark the median Distinct-1 for each role (patient = 0.435; interviewer = 0.329). Prompt-adherence failures are denoted by white or gray fill. White fill indicates a full prompt-adherence failure, while gray fill indicates a soft-failure, as described in Transcript Metrics in Results.

In attempting to generate 100 transcripts, 10 sessions (10%) failed to meet the stop-condition criterion established in the provided prompts, because the interview-assistant model either failed to output the required “<STOP>” token or ended the interview prematurely. This constitutes our measure of full prompt-adherence failure (See Figure 7, data points with white fill). A more subtle prompt-adherence issue (soft failure)—seen in both models—was the unintended disclosure of the full chain-of-thought process instead of the concise final answer due to improper formatting in the model's output (See Figure 7, data points with gray fill). When soft failure occurs, it typically dilutes lexical diversity and lowers the Distinct-1 score. When we restrict our analysis to only the inlier transcripts (i.e., excluding both full and soft failures), the median Distinct-1 scores remain essentially unchanged—0.444 for patient turns and 0.331 for assistant turns—confirming that prompt-adherence failures do not bias our diversity metrics. Regardless, transcripts flagged for prompt-adherence failures would not be included in finalized datasets used for downstream analyses (see Discussion 4.5).

An extension would be to perform a qualitative analysis using human volunteers and analysis of actual patient transcripts as ground truth. However, because of limitations around access to actual transcripts (and informed consent), such an approach is currently challenging. In lieu of a full expert study, we informally spot-checked several generated transcripts to ensure they read coherently, maintained empathic tone, and adhered to the prescribed interview flow. A systematic, blinded evaluation by clinical experts remains an important next step (see Discussion 4.5).

## 4 Discussion

The use of synthetic data for AI applications in mental health presents a promising avenue for addressing long-standing challenges related to data availability, privacy, and population representation (37). In this work, we introduce a framework for generating dynamic, demographically grounded, and narratively diverse synthetic psychiatry patients using Llama 3.3, a locally hosted and open-source large language model (38). By simulating psychiatric interviews between a virtual patient and an interview assistant model, our system provides a privacy-respecting and scalable alternative to traditional clinical datasets.

### 4.1 Role of synthetic data in AI for mental health

Clinical datasets in psychiatry are often limited by small sample sizes, unbalanced demographics, and strict privacy regulations (39–43). Synthetic patient generation offers a compelling alternative to these constraints by enabling the creation of realistic, representative, and reusable data without implication for patient confidentiality (44). Unlike de-identified clinical data, which can still pose re-identification risks, synthetic datasets generated from probabilistic models and language-based simulations eliminate direct links to real individuals (45–47).

As demonstrated in our demographic evaluations (Figure 4), the generated patients are constructed to align closely with real-world distributions in ethnicity, age, and disability type. By explicitly controlling for underrepresented characteristics, synthetic data can help mitigate biases that may go unaddressed in traditional datasets, especially in psychiatric contexts where social and cultural diversity significantly impact diagnosis and treatment or, at a more fundamental level, if an individual seeks care (37, 48).

Nevertheless, while synthetic patients offer strong utility for research and education, they cannot fully replace the complexity and nuance of real human experiences. Important aspects such as behavioral variability, hesitancy to discuss symptoms (stigma), subtle symptom progression, or comorbidities that evolve over time are not encompassed in a single interview. Synthetic patients and their interview transcripts are best viewed as a complement to real data that we must continue to improve as an instrument for model development, exploration, and testing. It will never be a full substitute for clinical interactions.

### 4.2 Comparison to existing AI approaches

Our approach diverges from AI systems trained on static datasets or structured clinical records. Domain-specific models like ClinicalBERT and Med-PaLM2 have demonstrated strong performance on predictive modeling, specifically prediction of hospital readmission, and medical question answering (US Medical Licensing Exam style-questions), respectively (49, 50). However, these systems are typically trained on datasets like MIMIC-III or MultiMedQA that contain limited interactive or psychiatric content (51, 52). While existing models have been successfully applied to structured medical tasks, their use in adaptive, context-dependent exchanges, an essential characteristic of psychiatric assessments, remains an area of ongoing exploration (53–59).

Existing synthetic data efforts have focused largely on structured tabular data, imaging, or Electronic Health Record (EHR) simulation, often using techniques such as Generative Adversarial Networks (GANs) or Variational Autoencoders (VAEs). While these methods have produced high-quality synthetic datasets for various medical domains (5, 60), work on interactive, narrative-based simulations is evolving rapidly. For example, other authors have used prompt engineering with ChatGPT to simulate patients and psychiatrists (61). Importantly their work shows that despite rational design of prompts to produce a more faithful clinical interaction, ultimately these models were rejected as artificial and disingenuous when evaluated by human users. Our contribution in this work is a framework producing full psychiatric interviews between LLMs acting in dialogue—one as the patient, the other as the interview assistant providing a test-bed for exploring AI tool development in mental health.

### 4.3 Applications and impact

Synthetic patients have several potential applications, notably in AI model validation, where they serve as standardized benchmarks for assessing conversational AI systems in psychiatry. The technique being developed whereby the machine generates demographically accurate synthetic responses that can be utilized in our broader vision for developing an AI agent capable of running structured interviews and ultimately more general psychiatric interviews that can provide time-saving summary documentation for clinical staff. For medical education, interactive synthetic interviews can provide trainees with realistic diverse scenarios, improving diagnostic reasoning and clinical interviewing skills.

Clinically, synthetic transcripts generated by large, computationally intensive models (e.g., Llama 3.3:70b, DeepSeek-R1, or proprietary state-of-the-art models) can be leveraged to fine-tune smaller, computationally efficient models suitable for broader deployment. While our current implementation using the Llama3.3:70b model operates within relatively modest hardware constraints, scaling up to accommodate many concurrent users would necessitate substantial hardware upgrades or cloud-based deployments –both of which introduce higher costs, complexity, and privacy concerns.

Instead, using high-quality synthetic datasets to fine-tune compact models enables the deployment of mental health chatbots or AI assistants even on modest infrastructure. Such smaller models can handle real-time interactions effectively, potentially transforming aspects of psychiatric care delivery by providing consistent initial assessments, augmenting therapeutic interactions, or serving as accessible mental health resources in underserved regions. Beyond the direct clinical potential being developed, the ability to create synthetic summaries that are accurate demographically to our population, and true-to-life can also be used in the educational setting and in clinical teaching. For example, they can be used by clinicians for creation of clinical vignettes in rounds, presentations, and examination settings which would be highly time-saving and improve diversity of clinical presentations being represented.

In the future, richer datasets could be generated from advanced open-source (e.g., DeepSeek-R1) or, licensing permitting, closed-source models (e.g., GPT-4o, GPT-4.5 or the Gemini models). This approach would provide broader narrative diversity and realism, further improving smaller models' conversational and potential diagnostic assistance.

### 4.4 Regulatory and ethical considerations

The development of AI-based mental health tools is constrained by strict privacy regulations, including the Health Insurance Portability and Accountability Act (HIPAA) in the United States, the General Data Protection Regulation (GDPR) in Europe, the Personal Information Protection and Electronic Documents Act (PIPEDA) in Canada, and British Columbia's Freedom of Information and Protection of Privacy Act (FIPPA), all which limit access to real patient data. Even when real-world data is available, it is often incomplete and may be insufficiently representative of the populations the AI system aims to serve. This makes data augmentation an essential strategy for mitigating bias and ensuring a more representative and comprehensive training dataset for any AI tool (60, 62).

With clinical datasets usually unavailable, this shifts the challenge to generating synthetic datasets that align with the characteristics of the intended population who will use the AI tool. To do so effectively, trusted data sources must be identified to inform the demographic and clinical distributions of synthetic patients. In the author's city, this includes publicly available datasets from Statistics Canada, the British Columbia Ministry of Education and Families, the British Columbia Ministry of Health, and the Vanier Institute of the Family. However, even these sources do not always provide data that is structured in a way that aligns with the specific details that practicing psychiatrists will request from their patients. In cases where key demographic or clinically relevant attributes (e.g., disability status, hospitalizations, family medical history) are missing, estimates are needed to approximate the distributions of certain characteristics. This process must be carefully documented alongside the synthetic generation code to ensure transparency and reproducibility. In this work, we have applied estimations for parameters relationship status by age and sex and the probability of having a child by age and sex with detailed rationals and probability estimates available in the GitHub repository (https://github.com/ubcbraincircuits/SPIT_Generation/tree/main/patient_creation/Estimation%20Rational). As recent reviews note, synthetic datasets must be critically assessed to ensure they do not perpetuate or amplify existing inequities (5). Continued iteration to improve estimates, incorporate community feedback, and engagement with potential end users will be essential for ensuring that synthetic mental health data is both ethically responsible and has the potential to improve clinical efficacy.

Although we have strived for demographic realism and transparency in constructing our synthetic patients, it is possible that bias may already be embedded in the statistical sources we have chosen. We emphasize that this system is in an early research stage and has not been applied to actual patients. Prior to any deployment outside of a research context, the project and its intended clinical uses would undergo full review by institutional ethics boards, including evaluation of the specific demographic data sources and statistical estimation procedures used to guide patient generation.

### 4.5 Challenges and future directions

Despite significant progress, challenges remain. As noted in the previous paragraph, developers of AI tools must remain mindful of the risk of bias propagation: assumptions underlying estimates must be understood and updated to avoid the generation of skewed synthetic datasets that perpetuate inaccuracies. Assessing the quality of psychiatric dialogue is another area which needs development. Although we included informal spot-checks of transcript realism (see Results 3.2.4), our evaluation remains largely focused around quantitative language metrics (Distinct-1 etc.). A systematic, blinded qualitative assessment by clinical experts will be essential to validate clinical tone, empathy, and symptom realism. While lexical diversity metrics like Duplicate Ratio and Distinct-1 (Figure 6) capture surface-level variation, more sophisticated tools are needed to assess clinical tone, empathy, and symptom realism including hesitancy on the part of the patient to discuss potentially stigmatizing symptoms. Additionally, prompt-adherence remains a practical challenge, as observed in our results (3.2.4, Figure 7), where both full and soft adherence failures occurred in a subset of generated transcripts. While such failures did not significantly bias lexical diversity measures, ongoing refinement of prompting strategies or automated detection methods will be essential to mitigate these issues and ensure dataset integrity. Furthermore, our approach inherits common risks associated with large language models, such as potential hallucinations (generation of factually incorrect or contextually inappropriate information) and inherent biases stemming from their training data. Although our structured prompting methods and demographic grounding reduce these risks, they cannot fully eliminate them. Future work should incorporate systematic monitoring for these issues and continue developing techniques, such as rigorous prompt engineering, automated content validation, and expert review, to further mitigate these risks. Finally, incorporating dataset-level validation could further enhance the robustness of the synthetic dataset. For example, one possible approach to reducing repetition in the LLM generated patient attributes could be the implementation of dataset-level validation techniques that dynamically adjust generated patient attributes based on real-time feedback from the model during generation, such as tracking all previously generated patients relaxation methods (yoga, meditation, etc.) within a generated dataset to ensure a variety of responses. Future work may also scale this framework to simulate broader domains such as psychological therapy, youth mental health, or cross-cultural care and continuous development is required to incorporate the benefits of improved foundation models.

## Data Availability

The original contributions presented in the study are included in the article/Supplementary Material, further inquiries can be directed to the corresponding author.
